# Analysis and experimental investigation of a subwavelength phased parallel-plate waveguide array for manipulation of electromagnetic waves

**DOI:** 10.1038/s41598-019-47272-8

**Published:** 2019-07-25

**Authors:** Dominic Palm, Zinching Dang, Marco Rahm

**Affiliations:** Department of Electrical and Computer Engineering, Research Center OPTIMAS, Technische Universität Kaiserslautern, Kaiserslautern, Germany

**Keywords:** Sub-wavelength optics, Micro-optics

## Abstract

Phase-gradient metasurfaces can be designed to manipulate electromagnetic waves according to the generalized Snell’s law. Here, we show that a phased parallel-plate waveguide array (PPWA) can be devised to act in the same manner as a phase-gradient metasurface. We derive an analytic model that describes the wave propagation in the PPWA and calculate both the angle and amplitude distribution of the diffracted waves. The analytic model provides an intuitive understanding of the diffraction from the PPWA. We verify the (semi-)analytically calculated angle and amplitude distribution of the diffracted waves by numerical 3-D simulations and experimental measurements in a microwave goniometer.

## Introduction

Phase shaping of electromagnetic waves has proven to be an effective approach for the implementation of a wealth of artificial electromagnetic devices such as electromagnetic beam steerers^1^, lenses^2–5^, holographs^6,7^ and electromagnetic cloaks^8,9^. It has been shown by *Yu, N. et al*.^10^ that diffraction from phase-gradient metasurfaces can be readily described by a generalized Snell’s law (GSL). The GSL predicts the angles under which an incident wave is diffracted from a phase-gradient metasurface in forward and backward direction, when the incidence angle of the wave is known. For incidence angles above the critical angle, the incident wave is partially diffracted into a surface wave that propagates along the metasurface^11^, while another fraction is deflected into higher diffraction orders. Especially for metasurfaces with periodic phase gradient, the reformulated GSL readily predicts the angular distribution of the diffraction orders dependent on the incidence angle of the wave^12^. Periodic phase gradient metasurfaces have been designed and implemented in the realms of electromagetnism^13,14^ and in the the acoustic regime^15–20^. Also dielectric nanostructures were used to provide phase gradients by waveguiding^21,22^. In this respect, new methods were evaluated for acoustic noise control^23^, unidirectional waveguiding^24^, cloaking^25^, metacages^26^, solar cells^27^ and leaky wave antennas^28^. By optimization, metasurfaces were devised to provide impedance matching for incident waves under an ultra-wide range of incidence angles and bianisotropic materials have been successfully used to exploit the full potential of phase-gradient metasurfaces for manipulation of waves^29–37^. For example, large-angle beam steering with superb efficiency was demonstrated by *Asadchy, V. S. et al*.^38^. Furthermore, theoretical techniques have been applied by *Epstein, A. et al*. to calculate reflection and transmission coefficients of Floquet modes on metasurfaces^39^.

While the GSL predicts the angles under which incident waves are deflected from a phase-gradient metasurface in forward and backward direction, it does not provide information about the intensity or amplitude distribution of the waves diffracted into the different orders. In order to obtain information about the amplitude distribution of the diffracted waves, either numerical calculations are required or an analytic model with the corresponding equation system must be derived and solved. Here, we consider a phased parallel-plate waveguide array (PPWA) rather than a phase-gradient metasurface. We show that a PPWA can be designed in the same manner as metasurfaces to provide functionalities described by the GSL. We develop an analytic model that fully describes the electromagnetic behavior of the PPWA and calculate both the diffraction angles and the amplitude distribution of diffracted waves from the PPWA in forward and backward direction by coupled mode theory. In addition, we deliver an intuitive model that explains why incident waves are only diffracted into specific reflected and transmitted diffraction orders. Furthermore, we compare the analytic description with 3-D full wave numerical simulations and provide physical insight into the coupling mechanism between the waves in the PPWA. Finally, we fabricate a PPWA according to the geometric parameters used in the numerical and analytic model and compare the calculated diffraction pattern with the measured diffraction pattern obtained in a microwave experiment.

## Theory

### Design

**Figure 1:**
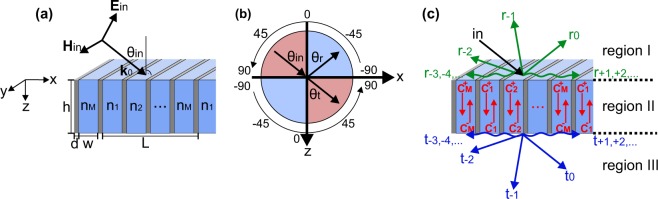
PPWA schematic, angle orientation and propagation modes. (**a**) Schematic of the PPWA. The waveguides are filled by impedance-matched material with refractive indices *n*_1_…*n*_*M*_. (**b**) Orientation of the angles of the incident (*θ*_*in*_), reflected (*θ*_*r*_) and transmitted (*θ*_*t*_) waves. (**c**) Schematic of the incident wave and the diffraction orders of the backward diffracted waves in region I, waveguide modes in region II and forward diffraction orders in region III.

As described in the previous section, we use a PPWA for tailoring the spatial phase of the waves that are transmitted through or reflected from the PPWA. A schematic of the PPWA is illustrated in Fig. 1(a). The array consists of a periodic arrangement of cells, each of which is comprised of *M* parallel-plate waveguides (PPWs). Hereby, the physical dimension of the waveguides are subwavelength with respect to the wavelength of the incident wave, while the (optical) size of the cells lies in the diffractive regime. Each cell has a width *L* and a length *h*. The width of the contained waveguides and the waveguide wall thickness are denoted by *w* and *d*, respectively. The material of the walls is assumed as perfect electric conductor.

The PPWs are individually filled by lossless material with refractive index *n*_*m*_, where *m* denotes the index of the PPW. We assume the material in the waveguides to be impedance-matched with vacuum, which implies that ${n}_{m}=\sqrt{{\varepsilon }_{m}{\mu }_{m}}$ and *ε*_*m*_ = *μ*_*m*_ where *ε*_*m*_ is the relative permittivity and *μ*_*m*_ is the relative permeability of the material in the *m*^th^ PPW. The refractive index is given by *n*_*m*_ = 1 + (*m* − 1)*λ*/*hM* with *h* = *λ*/(*n*_*max*_ − 1), where *λ* is the wavelength of the incident wave in free space and *n*_*max*_ is the maximal refractive index in the design. This refractive index distribution implies a spatial 2*π*-periodicity in the phase advance experienced by waves traveling from *z* = 0 to *z* = *h* through the *m*-th waveguide in neighboring cells.

For the remainder of the paper, we assume transverse magnetic polarization of the incident wave on the PPWA, as shown in Fig. 1(a). The incidence angle (*θ*_*in*_), reflection angle (*θ*_*r*_) and transmission angle (*θ*_*t*_) of the waves are defined with respect to the aperture normal of the PPWA. As shown in Fig. 1(b), angles are counted positive in the upper left quadrant and negative in the upper right quadrant, while they are defined negative in the lower left quadrant and positive in the lower right quadrant of the coordinate system. The sign definition allows a clear identification of the diffraction orders of diffracted waves from the PPWA in reflection and transmission direction.

### Analytic mode description

While the generalized Snell’s law predicts the directions under which waves are diffracted from a metasurface, it does not quantify the amplitude distribution of the diffracted waves^12,13^. For adequate description of the PPWA, we used coupled mode theory to calculate the dependence of the angular amplitude distribution of diffracted waves on the angle of the incidence wave^15,19^. We considered diffraction in both reflection and transmission direction of the PPWA. In more specific terms, we calculated the reflection and transmission coefficients of the PPWA in dependence on the incidence angle of the incoming wave.

For an analytic description, we divided the model into three distinct regions, as shown in Fig. 1(c). Region I expands from *z* = −∞ to *z* = 0 and from *x* = −∞ to *x* = +∞. It contains the electromagnetic fields of the incident waves and the fields that are diffracted back from the boundary at *z* = 0. In region II with $[0\le z\le h,-\,{\rm{\infty }}\le x\le +\,{\rm{\infty }}]$, the transmitted waves from region I travel in the PPWA in the  + *z*- and the −*z*-direction. Hereby, the waves in −*z*-direction originate from reflection at the boundary at *z* = *h*. Region III with $[h\le z\le \infty ,-\,\infty \le x\le +\,\infty ]$ includes the electromagnetic waves that are transmitted at *z* = *h* from the PPWA in region II into free space in region III.

Based on this model, we describe the electric and magnetic fields of the the incoming waves by

1 $${{\bf{E}}}_{in}=\exp ({\rm{i}}{k}_{x}x)\exp ({\rm{i}}{k}_{z}z){(\begin{array}{ccc}\frac{{k}_{z}}{{k}_{0}} & 0 & -\frac{{k}_{x}}{{k}_{0}}\end{array})}^{T}$$

2 $${{\bf{H}}}_{in}=\frac{1}{{\eta }_{I}}\exp ({\rm{i}}{k}_{x}x)\exp ({\rm{i}}{k}_{z}z){(\begin{array}{ccc}0 & 1 & 0\end{array})}^{T}$$

where *k*_0_ = 2*π*/*λ* denotes the absolute value of the wave vector of the incident wave in free space, *k*_*x*_ = sin(*θ*_*in*_)*k*_0_ the *x*-component of *k*_0_, ${k}_{z}=\sqrt{{k}_{0}^{2}-{k}_{x}^{2}}$ the *z*-component of *k*_0_ and *η*_*I*_ the wave impedance in region I.

The *l*^th^ order waves that are diffracted back into region I are described by the Floquet theorem corresponding to

3 $${{\bf{E}}}_{r,l}={r}_{l}\exp ({\rm{i}}{k}_{x,l}x)\exp (-{\rm{i}}{k}_{z,l}z){(\begin{array}{ccc}-\frac{{k}_{z,l}}{{k}_{0}} & 0 & -\frac{{k}_{x,l}}{{k}_{0}}\end{array})}^{T}$$

4 $${{\bf{H}}}_{r,l}=\frac{1}{{\eta }_{I}}{r}_{l}\exp ({\rm{i}}{k}_{x,l}x)\exp (-\,{\rm{i}}{k}_{z,l}z){(\begin{array}{ccc}0 & 1 & 0\end{array})}^{T}$$

where *k*_*x*,*l*_ = *k*_*x*_ + 2*πl*/*L* the *x*-component of the wave vector of the *l*^th^ order diffracted wave, ${k}_{z,l}=\sqrt{{k}_{0}^{2}-{k}_{x,l}^{2}}$ the *z*-component of the wave vector of the *l*^th^ order diffracted wave and *r*_*l*_ the reflection coefficient of the *l*^th^ order diffracted wave into region I.

The total electric and magnetic field in region I can be calculated by superposition of incident and back scattered fields as

5 $${{\bf{E}}}_{I}={{\bf{E}}}_{in}+\sum _{l=-\infty }^{+\infty }{{\bf{E}}}_{r,l}$$

6 $${{\bf{H}}}_{I}={{\bf{H}}}_{in}+\sum _{l=-\infty }^{+\infty }{{\bf{H}}}_{r,l}$$

In analogy, the fields that are diffracted into region III after propagation through the PPWA in region II can be expressed by

7 $${{\bf{E}}}_{t,l}={t}_{l}\exp ({\rm{i}}{k}_{x,l}x)\exp [{\rm{i}}{k}_{z,l}(z-h)]{(\begin{array}{ccc}\frac{{k}_{z,l}}{{k}_{0}} & 0 & -\frac{{k}_{x,l}}{{k}_{0}}\end{array})}^{T}$$

8 $${{\bf{H}}}_{t,l}=\frac{1}{{\eta }_{III}}{t}_{l}\exp ({\rm{i}}{k}_{x,l}x)\exp [{\rm{i}}{k}_{z,l}(z-h)]{(\begin{array}{ccc}0 & 1 & 0\end{array})}^{T}$$

9 $${{\bf{E}}}_{III}=\sum _{l=-\infty }^{+\infty }{{\bf{E}}}_{t,l}$$

10 $${{\bf{H}}}_{III}=\sum _{l=-\infty }^{+\infty }{{\bf{H}}}_{t,l}$$

where *t*_*l*_ denotes the transmission coefficient of the *l*^th^ order diffracted wave into region III and *η*_*III*_ indicates the wave impedance in region III. It should be noted that diffracted waves in region I and region III become evanescent for $|{k}_{x,l}| > {k}_{0}$ and thus propagate as confined surface waves along the boundary between region I and region II, respectively between region II and region III. Surface waves are depicted as curved arrows in Fig. 1(c).

For the analytic description of the field modes in the *m*^th^ waveguide of the PPWA, we exploit that the waveguide width *w* is very small compared with the wavelength of the propagating waves in the ±*z* direction. Under this condition, only the fundamental mode can propagate in the waveguides and the electric and magnetic field can be described by

11 $${{\bf{E}}}_{II,m}^{+}={C}_{m}^{+}\exp ({\rm{i}}{k}_{0}{n}_{m}z){(\begin{array}{ccc}1 & 0 & 0\end{array})}^{T}$$

12 $${{\bf{H}}}_{II,m}^{+}=\frac{1}{{\eta }_{II,m}}{C}_{m}^{+}\exp ({\rm{i}}{k}_{0}{n}_{m}z){(\begin{array}{ccc}0 & 1 & 0\end{array})}^{T}$$

13 $${{\bf{E}}}_{II,m}^{-}={C}_{m}^{-}\exp (\,-\,{\rm{i}}{k}_{0}{n}_{m}z){(\begin{array}{ccc}1 & 0 & 0\end{array})}^{T}$$

14 $${{\bf{H}}}_{II,m}^{-}=\frac{1}{{\eta }_{II,m}}{C}_{m}^{-}\exp (\,-\,{\rm{i}}{k}_{0}{n}_{m}z){(\begin{array}{ccc}0 & -1 & 0\end{array})}^{T}$$

where ${C}_{m}^{+}$ and ${C}_{m}^{-}$ are the complex amplitudes of the fundamental mode in the *m*^th^ waveguide. The (+)-sign in the indices refers to propagation in +*z*-direction, while the (−)-sign indicates propagation in −*z*-direction. *η*_*II*,*m*_ denotes the wave impedance in the *m*^th^ waveguide. The total electric and magnetic field in the *m*^th^ waveguide is a linear superposition of the propagating modes according to

15 $${{\bf{E}}}_{II,m}={{\bf{E}}}_{II,m}^{+}+{{\bf{E}}}_{II,m}^{-}$$

16 $${{\bf{H}}}_{II,m}={{\bf{H}}}_{II,m}^{+}+{{\bf{H}}}_{II,m}^{-}$$

To solve our equation system, we must express the boundary conditions for *E*_*x*_ and *H*_*y*_ at *z* = 0 and *z* = *h* for each waveguide in the PPWA. For a detailed calculation, we refer to Supplementary Information, chapter 1.

From the boundary conditions, we obtain

17 $${r}_{l}=\frac{{\delta }_{0l}{k}_{z}-\frac{w}{L}\sum _{m=1}^{M}({C}_{m}^{+}+{C}_{m}^{-}){A}_{l,m}^{-}{k}_{0}}{{k}_{z,l}}$$

18 $$\sum _{l=-\infty }^{+\infty }{r}_{l}{A}_{l,m}^{+}=\frac{{\eta }_{I}}{{\eta }_{II,m}}({C}_{m}^{+}-{C}_{m}^{-})-{A}_{\mathrm{0,}m}^{+}$$

19 $${t}_{l}=\frac{w}{L}\frac{{k}_{0}}{{k}_{z,l}}\sum _{m=1}^{M}[{C}_{m}^{+}\exp ({\rm{i}}{k}_{0}{n}_{m}h)+{C}_{m}^{-}\exp (\,-\,{\rm{i}}{k}_{0}{n}_{m}h)]{A}_{l,m}^{-}$$

20 $$\sum _{l=-\infty }^{+\infty }{t}_{l}{A}_{l,m}^{+}=\frac{{\eta }_{III}}{{\eta }_{II,m}}[{C}_{m}^{+}\exp ({\rm{i}}{k}_{0}{n}_{m}h)-{C}_{m}^{-}\exp (\,-\,{\rm{i}}{k}_{0}{n}_{m}h)]$$

$${\rm{w}}{\rm{i}}{\rm{t}}{\rm{h}}\,{A}_{l,m}^{\pm }=\exp [\,\pm \,{\rm{i}}{k}_{x,l}(m-1)(w+d)]{\rm{s}}{\rm{i}}{\rm{n}}{\rm{c}}\frac{w{k}_{x,l}}{2}$$

where *δ*_0*l*_ is the Kronecker delta and sinc(*x*) = sin(*x*)/*x* is the unnormalized sinc function.

By solving the equation system (17) to (20), we obtain the reflection coefficient *r*_*l*_ and the transmission coefficient *t*_*l*_ as well as the amplitudes ${C}_{m}^{+}$ and ${C}_{m}^{-}$ of the modes in the waveguides. For adequate consideration of the relative intensities of the waves in the different diffraction orders, we normalized the transmitted and reflected intensities by the factors *cosθ*_*t*_/*cosθ*_*in*_ and *cosθ*_*r*_/*cosθ*_*in*_, respectively. Hereby, *θ*_*in*_ represents the incidence angle of the wave, ${\theta }_{t}={sin}^{-1}({k}_{x,l}/{k}_{0})$ and ${\theta }_{r}={\sin }^{-1}(\,-\,{k}_{x,l}/{k}_{0})$ the propagation angle of the transmitted, respectively of the reflected wave in the *l*^th^ diffraction order.

## Results and Discussion

### Comparison between the analytic model and numerical calculations

In order to test the validity of our analytic model, we chose an arbitrary PPWA configuration and numerically calculated the electric and magnetic fields of waves that are diffracted from the PPWA. For comparison, we calculated the propagation angles and the electric and magnetic field amplitude of the waves in the different diffraction orders by means of the analytic description. As an example, we chose a PPWA with 6 waveguides per unit cell and a ratio between wavelength and cell width of *λ*/*L* = 0.8. It is notable that the diffractive properties of the PPWA in back and forward direction are independent of the wave frequency as long as the ratios between the wavelength *λ*, the waveguide width *w*, the waveguide wall thickness *d* and the waveguide length *h* are constant. For later experimental verification in a microwave goniometer, we evaluated the analytic and numerical model for a wave frequency of 10.41 GHz, which corresponds to a wavelength of 28.9 mm in free space. For the maximal refractive index in the design we chose ${n}_{max}=\sqrt{2.1}$. The width of the unit cell was *L* = 36 mm, the waveguide width *w* = 5 mm, the wall thickness *d* = 1 mm and the length of the waveguide *h* = 64.1 mm.

**Figure 2:**
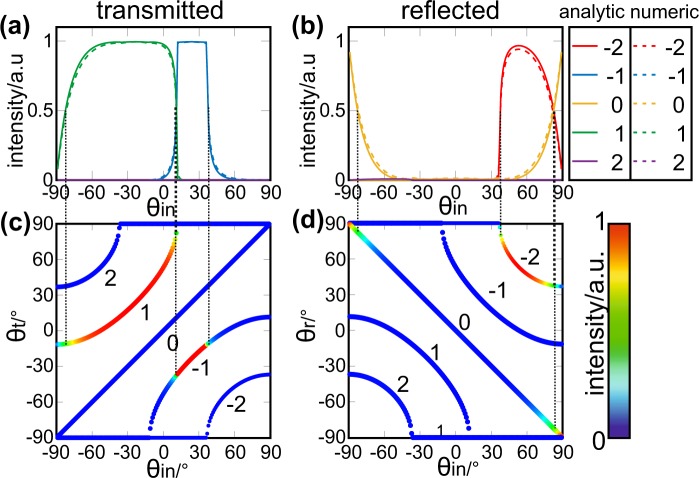
Intensity distribution and angular dependence of the diffraction orders of the PPWA (**a**,**b**) Analytically and numerically calculated dependence of the intensity of the forward and backward diffracted wave intensities from the PPWA for orders from −2 to +2. The analytic and numerical results are in excellent agreement. (**c**,**d**) Dependence of the angle distribution of the forward (*θ*_*t*_) and backward (*θ*_*r*_) diffracted waves on the incidence angle *θ*_*in*_ together with the angle-dependent intensity distribution of the waves in the different diffraction orders.

Figure 2 shows the dependence of the intensity transmissivity (Fig. 2(a)) and reflectivity (Fig. 2(b)) of the diffracted waves from the PPWA on the incidence angle for diffraction orders from *l* = −2 to +2. Although we also calculated the transmittivity and reflectivity for diffraction orders *l* = −10 to +10, results for |*l*| > 2 are not displayed in Fig. 2, since waves in those diffraction orders are evanescent for all incidence angles. As a result, they propagate as bound surface waves in *x*-direction along the waveguide apertures and do not radiate into free space. The calculated intensity transmissivity and reflectivity of the waves in diffraction orders *l* = −2 to +2 are also plotted in the color maps of Fig. 2(c,d). The color maps provide simultaneous information about the dependence of the diffraction angle on the incidence angle of the waves and the intensity transmissivity and reflectivity into the different diffraction orders.

For incidence angles −90° < *θ*_*in*_ < −80° according to their definition in Fig. 1(b), more than 50% of the incident wave intensity is reflected into the diffraction order *l* = 0, see Fig. 2(b,d), while less than 50% of the wave intensity is transmitted into the diffraction order *l* = +1 according to Fig. 2(a,c). For smaller negative incidence angles −80° <  *θ*_*in*_ < *θ*_*crit*_ ≈ 11.5°, the transmissivity into the diffraction order *l* = +1 increases to 1 and the reflection into the diffraction order *l* = 0 vanishes. In this regime, the transmitted wave is deflected in *x*-direction due to the phase gradient imposed on the propagating wave during transmission through the PPWA. At the critical incidence angle, the transmitted wave in the *l* = +1 diffraction order propagates at an angle of exactly 90° to the *z*-axis and thus becomes evanescent. As a consequence, the transmission into the *l* = +1 diffraction order becomes zero as soon as the incidence angle exceeds the critical angle. Our analytic calculation shows that the transmitted wave is completely diffracted into the *l* = −1 diffraction order for incidence angles *θ*_*crit*_ < *θ*_*in*_ < 37°, while the reflectivity remains zero. For incidence angles 37° < *θ*_*in*_ < 80°, the reflection into the *l* = −2 diffraction order abruptly increases and the transmission into the *l* = −1 order decreases until all incident radiation is reflected into the *l* = −2 and *l* = 0 order. As can be seen from Fig. 1(b), the reflectivity into the *l* = 0 order is symmetric with respect to the incidence angle.

Before providing a physical explanation for the dependence of the intensity distribution between the different diffraction orders in reflection and transmission, we validate the analytic results by comparing the calculated transmissivity and reflectivity and the diffraction angles with the corresponding quantities obtained from numerical calculations using CST Microwave Studio. In the numerical model, we defined the calculation domain by unit cell boundaries and applied Floquet ports with the same diffraction orders *l* = −10 to *l* = +10 as in the analytic calculation. The numerically calculated transmissivity, reflectivity as well as the diffraction angle dependence on the incidence angle are plotted in Fig. 2(a) to 2(d) as dashed lines and are in excellent agreement with the analytic results.

**Figure 3:**
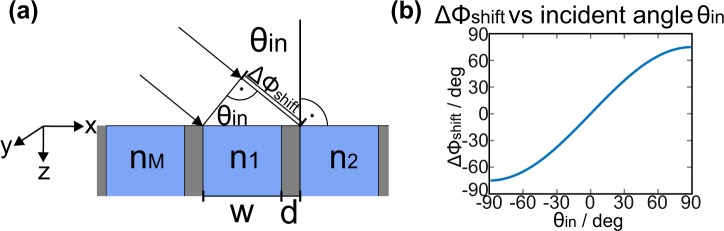
Angle-dependent spatial phase shift of the incident waves. (**a**) Schematic of the geometrically evaluated spatial phase shift of the wave, which depends on the incidence angle. The spatial phase shift Δ*ϕ*_shift_ is calculated as the difference between the spatial phase of the wave at the left and right wall of a waveguide of width *w* at position *z* = 0. (**b**) Spatial phase shift Δ*ϕ*_shift_ vs. incidence angle *θ*_in_ of the wave.

With the aim of providing a more intuitive and physical explanation of the diffractive behavior of the PPWA below and above the critical angle of incidence, we analyzed the amplitude and phase advance of the waves in the waveguides and at the respective boundaries. In this context, we must also account for the initial phase of the waves at the boundaries between region I and region II that solely depends on the angle of incidence. As illustrated in Fig. 3(a), the relative phase difference between waves at the entrance of neighboring waveguides at *z* = 0 can be calculated based on geometrical considerations as Δ*ϕ*_*shift*_ = sin(*θ*_*in*_)(*w* + *d*)2*π*/*λ*. The quantitative dependence of Δ*ϕ*_*shift*_ on the incidence angle *θ*_*in*_ is plotted in Fig. 3(b). In order to analyze the influence of the amplitude and phase advance of the waves in the waveguides on the diffractive properties of the PPWA, it is instructive to compensate for the dependence of the initial phase on the incidence angle by subtracting a value of (*m* + *iM*)Δ*ϕ*_*shift*_ at *z* = 0 for each waveguide. Hereby, *i* is the number of the considered PPWA cell and *m* is the index of the waveguide in the *i*^th^ cell of the PPWA. In the following, we refer to such waveguide modes as phase-compensated modes.

**Figure 4:**
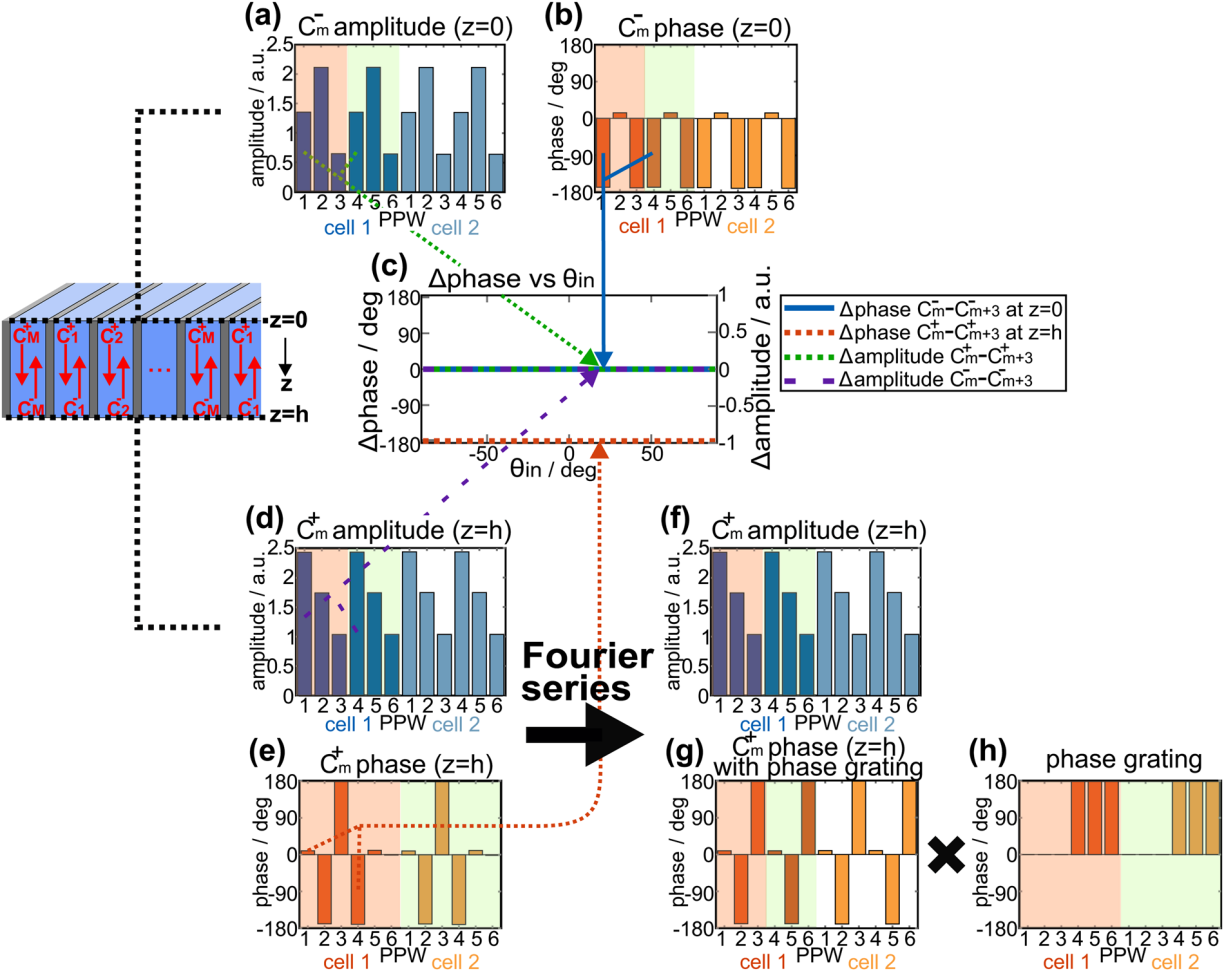
Complex ${C}_{m}^{+}$ and ${C}_{m}^{-}$ of the phase compensated waveguide modes. (**a**,**b**) Amplitude and compensated phase of ${C}_{m}^{-}$ at *z* = 0 for the example of two neighboring cells, each with a width *L*, corresponding to 6 waveguides in a cell. The phase is compensated for an incident wave at an angle of 21°. The red and green area highlight the periodicity *L*/2 of the amplitude and phase distribution. The periodicity is on par with exactly three cells. (**c**) Amplitude and phase difference of ${C}_{m}^{+}-{C}_{m+3}^{+}$ and ${C}_{m}^{-}-{C}_{m+3}^{-}$ at *z* = 0 and *z* = *h*, respectively. (**d**,**f**) Amplitude of ${C}_{m}^{+}$ at *z* = *h* for the example of two neighboring cells. The phase is compensated for an incident wave at an angle of 21°. The red and green area highlight the periodicity *L*/2 of the amplitude distribution. The periodicity is on par with exactly three cells. (**e**) Compensated phase of ${C}_{m}^{+}$ at *z* = *h* for an incident wave at an angle of 21°. The periodicity of the phase is *L*, which corresponds to exactly six waveguides. (**g**,**h**) The compensated phase of ${C}_{m}^{+}$ at *z* = *h* can be interpreted as a superposition of an *L*/2-periodic phase distribution, (**g**), and an *L*-periodic binary phase grating, (**h**).

First, we analyze the back diffraction at *z* = 0 for the compensated waveguide modes that are described by the phase-compensated complex amplitude ${C}_{m}^{-}$. Figure 4(a,b) show an excerpt of the amplitude and (compensated) phase of ${C}_{m}^{-}$ at *z* = 0 in two cells of the PPWA for the example of an incidence angle of 21°. As can be seen from the plots, both the amplitude and the (compensated) phase of ${C}_{m}^{-}$ at *z* = 0 are periodic with a periodicity of *L*/2, where *L* is the width of a cell. In other words, the PPWA acts like an amplitude and phase grating in reflection direction with an effective periodicity of *L*/2. This is an interesting finding, since the PPWA with a spatial periodicity of *L* obviously impregnates a periodic electromagnetic modulation on the waves with an effective periodicity that is only half of the spatial periodicity of the PPWA. With respect to the spatial periodicity *L*, we therefore only observe back diffraction into even numbered orders ($\ldots ,-4,-2,\,0,+2,+4,\ldots$), according to a grating contribution of $n\cdot \frac{2\pi }{(L\mathrm{/2)}}=2n\cdot \frac{2\pi }{L}$ for ($n=\mathrm{0,}\pm \,\mathrm{1,}\pm \,\mathrm{2,}\ldots$). This behavior can be observed for all wave incidence angles between −89° and 89°. In this respect, Fig. 4(c) depicts the dependence of the amplitude and (compensated) phase difference ${C}_{m+3}^{-}-{C}_{m}^{-}$ within a cell on the incidence angle. Again, *m* denotes the waveguide number in the cell. As explained above, we observe that the amplitude and phase difference of ${C}_{m+3}^{-}-{C}_{m}^{-}=0$ equals zero for all incidence angles, which evidences the periodicity of *L*/2 for the wave modulation in the PPWA. The observation of back diffraction into solely even numbered diffraction orders for all incidence angles is in perfect agreement with the numerical and analytic calculations of the diffraction orders, as shown in Fig. 2(d).

Second, we study the diffraction of the waves at the boundary between the PPWA and free space at *z* = *h*. The diffracted waves are described by the phase-compensated complex amplitude ${C}_{m}^{+}$. The amplitude and phase of ${C}_{m}^{+}$ at *z* = *h* are plotted in Fig. 4(d,e) for the example of a wave incidence angle of 21°. While the amplitude of ${C}_{m}^{+}$ within a cell is periodically modulated with a periodicity of *L*/2 (Fig. 4(d)), the (compensated) phase of ${C}_{m}^{+}$ changes periodically with a periodicity of *L* (Fig. 4(e)). It is peculiar that the phase of ${C}_{m}^{+}$ of waves in waveguides in the second half period of a cell is shifted by 180° with respect to the phase of ${C}_{m}^{+}$ of waves in waveguides in the first half period of the same cell. A calculation of ${C}_{m+3}^{-}-{C}_{m}^{-}$ within a cell lets us conclude that an identical amplitude and phase modulation can be observed for all incidence angles between −89° and 89°.

An intuitive approach to describe the observed 180° shift in the phase of ${C}_{m}^{+}$ every half cycle is to picture the (compensated) phase of ${C}_{m}^{+}$ as the result of a superposition of an *L*/2-periodic amplitude and phase distribution *g*(*x*), as in Fig. 4(f,g), and a phase grating function *h*(*x*) that induces an *L*-periodic binary phase shift according to Fig. 4(h). In other words, the complex ${C}_{m}^{+}$, i.e. the amplitude and compensated phase of ${C}_{m}^{+}$, can be described as the product of the *L*/2-periodic amplitude and phase distribution *g*(*x*) and an *L*-periodic binary phase grating function *h*(*x*). Both functions *g*(*x*) and *h*(*x*) can be described by a Fourier series according to

21 $$g(x)=\sum _{k=-\infty }^{+\infty }{a}_{k}\exp ({\rm{i}}\,k4\pi /Lx)$$

22 $$h(x)=\sum _{k^{\prime} =-\infty }^{+\infty }\frac{-2{\rm{i}}}{k^{\prime} \pi }{\rm{e}}{\rm{x}}{\rm{p}}[{\rm{i}}\,\mathrm{(2}k^{\prime} +\mathrm{1)}\,2\pi /Lx]$$

with $k,k^{\prime} \in {\mathbb{Z}}$ and *a*_*k*_ is a complex Fourier amplitude. For the product *f*(*x*) = *g*(*x*) × *h*(*x*), we obtain

23 $$f(x)=g(x)\times h(x)=\sum _{k^{\prime} =-\infty }^{+\infty }\sum _{k=-\infty }^{+\infty }\frac{-2{\rm{i}}{a}_{k}}{k^{\prime} \pi }\exp \{{\rm{i}}\,\mathrm{[2(}k^{\prime} +k)+\mathrm{1]2}\pi /Lx\}$$

Details on the Fourier series expansion are described in Supplementary Information, chapter 2.

As can be seen from Eq. (23), the PPWA acts as an amplitude and phase grating that only diffracts transmitted waves into odd-numbered diffraction orders [2(*k*′ + *k*) + 1] with respect to the reciprocal (one-dimensional) grating vector 2*π*/*L*. This result agrees with the analytic and numerical calculations of the diffraction of waves after transmission through the PPWA, illustrated in Fig. 2(c). Those calculations also indicate that waves are only transmitted into odd-numbered diffraction orders ($\ldots ,-\,\mathrm{3,}-\,\mathrm{1,}+\,\mathrm{1,}+\,\mathrm{3,}\ldots$) for all incidence angles.

A similar Fourier approach to describe diffraction from a periodic surface was used e.g. in^40–42^.

### Experimental results

**Figure 5:**
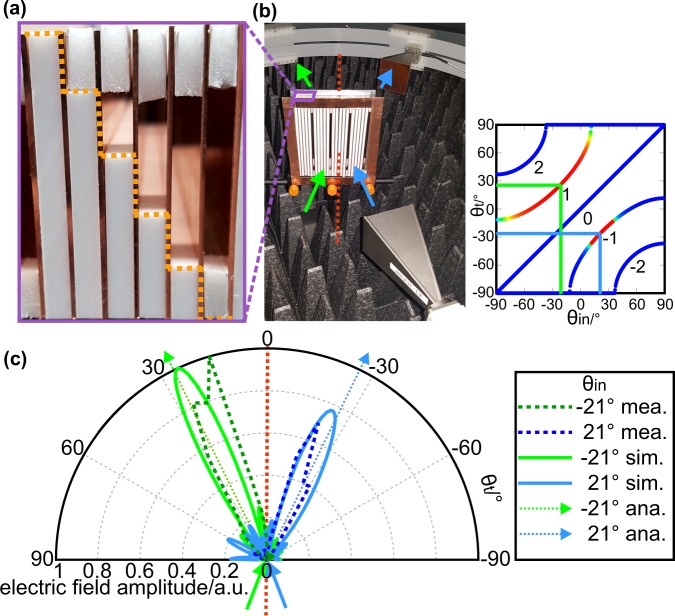
Implemented PPWA and measurement of the angular diffraction. (**a**) Top view of a cell of the PPWA. The PPWA is loaded with PTFE of different length to generate a phase gradient for waves propagating in adjacent waveguides. As additional spacers we used Airplac FIXTIC. (**b**) Microwave setup for measuring the angular diffraction distribution of the PPWA. The green and blue arrow indicate an incidence angle of −21°, which is below the critical angle, and +21°, which lies above the critical angle. (**c**) Polar chart of the measured (dashed line), numerically simulated (solid line) and analytically calculated (dotted line) angular amplitude distribution of the transmitted microwave electric field for an incidence angle of −21° (green lines) and +21° (blue lines). The analytically calculated diffraction angles are denoted by the dotted arrows for the corresponding incidence angles.

For the experimental verification of the analytic calculations, we implemented a PPWA with 4 unit cells along the *x*-direction. Each unit cell consisted of 6 PPWs with a waveguide width *w* = 5 mm, wall thickness *d* = 1 mm (both in *x*-direction) and a waveguide length *h* = 53 mm (in *z*-direction) as described in Fig. 1(a). The total aperture of the PPWA was 145 mm (aperture width in *x*-direction) × 160 mm (aperture height in *y*-direction). In our analytic model, we imposed a phase gradient on waves traveling through neighboring PPWs by filling the PPWs with impedance-matched material of different refractive index. In the fabricated PPWA however, we implmented the necessary phase gradient by loading each individual PPW with the similar non-impedance-matched medium, but varied the filling factor to control and change the optical path length in the different PPWs. In order to minimize reflection and loss from the filling material, we used Polytetrafluoroethylene (PTFE) as low-loss dielectric with a loss tangent tan*δ* = 0.0002^43^ and a dielectric constant *ε*_*diel*_ = 2.1 at a frequency of 10.41 GHz. The PPWs, numbered from 1 to 6, were filled by PTFE with a depth along the wave propagation direction of 0 mm, 11 mm, 21 mm, 32 mm, 43 mm, and 53 mm. The variation of the filling factor of the PPWs can be seen in the top view photograph of the unit cell in Fig. 5(a), which is emphasized by the orange dashed line. In addition, we used small pieces of Airplac as stabilizing spacers at the exit aperture of the PPWs. Since the refractive index of Airplac FIXTIC is almost identical to the refractive index of air, the material does not influence the propagation of the waves in the PPWs.

We measured the angle-dependent transmission of the PPWA at its working frequency of 10.41 GHz by means of a Vector Network Analyzer (Keysight FieldFox N9918A) and a microwave goniometer in an anechoic chamber, as shown in Fig. 5(b). The aperture of the emitting horn antenna of the goniometer was located at a fixed distance of 280 mm from the center point of the PPWA. To measure the angular dependence of the electric field amplitude of the diffracted waves, we moved the receiving horn antenna on a semicircle with a radius of 280 mm, measured as the distance between the center point of the PPWA and the aperture of the receiving horn antenna. We investigated the transmitted electric field distribution of the diffracted microwave field for two incidence angles, one at −21° below the critical angle, and the other at +21° above the critical angle. Since the measured and numerically calculated absolute electric fields of the diffracted waves differ due to non-determinable differences in the excitation power in the experiment and the calculations, we normalized all electric field amplitudes to the maximum of the electric field amplitude of the diffracted wave for an incidence angle of −21°. By that means, we were able to compare the relative angular electric field distribution of the diffracted waves in the measurement and the numerical simulation separately. Because the analytically determined diffraction fields were calculated under neglect of material loss and under the assumption of a PPWA with infinite aperture, we only computed the discrete diffraction direction and added it to the polar chart in Fig. 5(c) without declaration of the amplitude distribution. Due to an assumed infinite aperture and plane wave excitation in the analytic model, the diffraction in the analysis occurs at discrete angles, while the finite waves in the experiment and the numerical calculations diffract into diffraction lobes. Figure 5(c) depicts the polar chart of the normalized measured, the normalized numerically calculated angular amplitude distribution as well as the analytically calculated direction of the diffracted microwave electric field for the incidence angles of −21° (green lines) and +21° (blue lines). The green and blue arrows in the lower hemisphere indicate the direction of the incident radiation for the incidence angle of −21° and +21°. For the numerical calculations, we used the time domain solver of CST with open boundary conditions. The analytically and numerically calculated directions for the diffraction maxima are in perfect agreement. Also the measured angular distribution of the diffracted microwave electric field fits well with the theoretical calculations. Slight deviations between measurement results and theoretical results can be explained in terms of fabrication tolerances in the PPWA and systematic uncertainties in the measurement setup. The differences between the shape of the main and side lobes in the measurements and the numerical calculations may originate from the fact that the calculations assumed perfect plane waves for the incident microwaves. However, in the measurement, the incident microwaves were emitted by a horn antenna and the diffracted waves were measured by a second horn antenna, each at a distance of 280 mm from the center point of the PPWA. This implies that the wavefronts of the diffracted waves are measured in the far field (~10*λ*) and are not perfectly plane. Nevertheless, as predicted, microwaves are diffracted into the diffraction order *l* = +1 for an incidence angle below the critical angle. For an incidence angle above the critical angle, microwaves are transmitted into the diffraction order *l* = −1. During the angle scan, we did not observe any transmission into the diffraction order *l* = 0, which agrees with the theoretical predictions.

## Conclusion

We demonstrated a phased parallel-plate waveguide array (PPWA) that behaves equivalent to a phase gradient metasurface according to the generalized Snell’s law. We developed an analytic model to describe the wave propagation in the PPWA and calculated both the angle and amplitude distribution of the forward and backward diffracted waves from the PPWA by means of coupled mode theory. Based on the analytic equations, we found an intuitive explanation for the diffraction of the waves into the different orders and understood the dependence of the diffraction angle on the incidence angle of the waves. In addition, we numerically calculated the electromagnetic fields inside and outside the PPWA and observed excellent agreement between the analytically and numerically obtained field distributions. Using a microwave goniometer, we confirmed that the theoretically predicted forward diffraction angles of the PPWA agree with the measured ones.

## Supplementary information

Supplementary Information: Analysis and experimental investigation of a subwavelength phased parallel-plate waveguide array for manipulation of electromagnetic waves

## References

[1] Tsai Y-J (2011). Design and fabrication of a metamaterial gradient index diffraction grating at infrared wavelengths. Optics Express.

[2] Verslegers L (2009). Planar Lenses Based on Nanoscale Slit Arrays in a Metallic Film. Nano Letters.

[3] Paul O, Reinhard B, Krolla B, Beigang R, Rahm M (2010). Gradient index metamaterial based on slot elements. Applied Physics Letters.

[4] Aieta F (2012). Aberration-Free Ultrathin Flat Lenses and Axicons at Telecom Wavelengths Based on Plasmonic Metasurfaces. Nano Letters.

[5] Zhu W (2015). A Flat Lens with Tunable Phase Gradient by Using Random Access Reconfigurable Metamaterial. Advanced Materials.

[6] Huang L (2013). Three-dimensional optical holography using a plasmonic metasurface. Nature Communications.

[7] Zheng G (2015). Metasurface holograms reaching 80% efficiency. Nature Nanotechnology.

[8] Ni X, Wong ZJ, Mrejen M, Wang Y, Zhang X (2015). An ultrathin invisibility skin cloak for visible light. Science.

[9] Estakhri NM, Alu A (2014). Ultra-Thin Unidirectional Carpet Cloak and Wavefront Reconstruction With Graded Metasurfaces. IEEE Antennas and Wireless Propagation Letters.

[10] Yu N (2011). Light Propagation with Phase Discontinuities: Generalized Laws of Reflection and Refraction. Science.

[11] Sun S (2012). Gradient-index meta-surfaces as a bridge linking propagating waves and surface waves. Nature Materials.

[12] Xie Y (2014). Wavefront modulation and subwavelength diffractive acoustics with an acoustic metasurface. Nature Communications.

[13] Xu Y, Fu Y, Chen H (2015). Steering light by a sub-wavelength metallic grating from transformation optics. Scientific Reports.

[14] Cheng J, Inampudi S, Mosallaei H (2017). Optimization-based Dielectric Metasurfaces for Angle-Selective Multifunctional Beam Deflection. Scientific Reports.

[15] Mei J, Wu Y (2014). Controllable transmission and total reflection through an impedance-matched acoustic metasurface. New Journal of Physics.

[16] Liu B, Zhao W, Jiang Y (2016). Apparent Negative Reflection with the Gradient Acoustic Metasurface by Integrating Supercell Periodicity into the Generalized Law of Reflection. Scientific Reports.

[17] Liu B, Zhao J, Xu X, Zhao W, Jiang Y (2017). All-angle Negative Reflection with An Ultrathin Acoustic Gradient Metasurface: Floquet-Bloch Modes Perspective and Experimental Verification. Scientific Reports.

[18] Fang Y, Zhang X, Zhou J (2017). Sound transmission through an acoustic porous metasurface with periodic structures. Applied Physics Letters.

[19] Liu B, Jiang Y (2018). Metasurface-based angle-selective multichannel acoustic refractor. Applied Physics Express.

[20] Shen C, Cummer SA (2018). Harnessing Multiple Internal Reflections to Design Highly Absorptive Acoustic Metasurfaces. Physical Review Applied.

[21] Khorasaninejad M (2016). Polarization-Insensitive Metalenses at Visible Wavelengths. Nano Letters.

[22] Guo Zhongyi, Tian Lihua, Shen Fei, Zhou Hongping, Guo Kai (2017). Mid-infrared polarization devices based on the double-phase modulating dielectric metasurface. Journal of Physics D: Applied Physics.

[23] Wang X, Yu W, Jiang Z, Wang X, Mao D (2019). Acoustic gradient surfaces and gradient-index surfaces: Principles and applications on noise control. Applied Acoustics.

[24] Zhu Y-F, Zou X-Y, Liang B, Cheng J-C (2015). Broadband unidirectional transmission of sound in unblocked channel. Applied Physics Letters.

[25] Zhang J, Lei Mei Z, Ru Zhang W, Yang F, Jun Cui T (2013). An ultrathin directional carpet cloak based on generalized Snell’s law. Applied Physics Letters.

[26] Shen C, Xie Y, Li J, Cummer SA, Jing Y (2018). Acoustic metacages for sound shielding with steady air flow. Journal of Applied Physics.

[27] Khan MR, Wang X, Bermel P, Alam MA (2014). Enhanced light trapping in solar cells with a meta-mirror following generalized Snell’s law. Optics Express.

[28] Fan Y (2018). Frequency Scanning Radiation by Decoupling Spoof Surface Plasmon Polaritons via Phase Gradient Metasurface. IEEE Transactions on Antennas and Propagation.

[29] Wong JPS, Epstein A, Eleftheriades GV (2016). Reflectionless Wide-Angle Refracting Metasurfaces. IEEE Antennas and Wireless Propagation Letters.

[30] Asadchy VS (2016). Perfect control of reflection and refraction using spatially dispersive metasurfaces. Physical Review B.

[31] Epstein A, Eleftheriades GV (2016). Synthesis of Passive Lossless Metasurfaces Using Auxiliary Fields for Reflectionless Beam Splitting and Perfect Reflection. Physical Review Letters.

[32] Asadchy VS (2017). Flat Engineered Multichannel Reflectors. Physical Review X.

[33] Díaz-Rubio A, Asadchy VS, Elsakka A, Tretyakov SA (2017). From the generalized reflection law to the realization of perfect anomalous reflectors. Science Advances.

[34] Díaz-Rubio A, Tretyakov SA (2017). Acoustic metasurfaces for scattering-free anomalous reflection and refraction. Physical Review B.

[35] Lavigne G, Achouri K, Asadchy VS, Tretyakov SA, Caloz C (2018). Susceptibility Derivation and Experimental Demonstration of Refracting Metasurfaces Without Spurious Diffraction. IEEE Transactions on Antennas and Propagation.

[36] Chen M, Abdo-Sánchez E, Epstein A, Eleftheriades GV (2018). Theory, design, and experimental verification of a reflectionless bianisotropic Huygens’ metasurface for wide-angle refraction. Physical Review B.

[37] Li J, Shen C, Díaz-Rubio A, Tretyakov SA, Cummer SA (2018). Systematic design and experimental demonstration of bianisotropic metasurfaces for scattering-free manipulation of acoustic wavefronts. Nature Communications.

[38] Asadchy VS, Wickberg A, Díaz-Rubio A, Wegener M (2017). Eliminating Scattering Loss in Anomalously Reflecting Optical Metasurfaces. ACS Photonics.

[39] Epstein A, Eleftheriades GV (2014). Floquet-Bloch analysis of refracting Huygens metasurfaces. Physical Review B - Condensed Matter and Materials Physics.

[40] Cui TJ (2014). Coding metamaterials, digital metamaterials and programmable metamaterials. Light: Science and Applications.

[41] Kim M, Jeong J, Poon JKS, Eleftheriades GV (2016). Vanadium-dioxide-assisted digital optical metasurfaces for dynamic wavefront engineering. J. Opt. Soc. Am. B.

[42] Wong AM, Eleftheriades GV (2018). Perfect Anomalous Reflection with a Bipartite Huygens’ Metasurface. Physical Review X.

[43] Material libary. *CST Studio Suite 2018* (2018).

