# Supplementary material for: Analysis and experimental investigation of a subwavelength phased parallel-plate waveguide array for manipulation of electromagnetic waves
Source: Sci Rep. 2019 Jul 25;9:10792. doi: 10.1038/s41598-019-47272-8 (PMC6658516; doi:10.1038/s41598-019-47272-8)
Supplement: Supplementary file 1 — Supplementary Information: Analysis and experimental investigation of a subwavelength phased parallel-plate waveguide array for manipulation of electromagnetic waves [file 41598_2019_47272_MOESM1_ESM.pdf]

Supplementary Information: Analysis and experimental investigation of a subwavelength phased parallel-plate waveguide array for manipulation of electromagnetic waves

Dominic Palm[1,*], Zinching Dang[1], and Marco Rahm[1]

[1]Department of Electrical and Computer Engineering, Research Center OPTIMAS, Technische Universität Kaiserslautern, Kaiserslautern, Germany
[*]dpalm@eit.uni-kl.de

This document provides supplementary information to "Analysis and experimental investigation of a subwavelength phased parallel-plate waveguide array for manipulation of electromagnetic waves". Chapter 1 details the derivation of the equation system for the analytic description of the phased parallel-plate waveguide array (PPWA) (Eq. (17) - (20) in the main article) based on the boundary conditions for E_x and H_y at $z = 0$ and $z = h$. Chapter 2 describes the Fourier series expansion of the compensated waveguide modes in the PPWA that are used to explain the angular dependence of the back and forward diffraction induced by the PPWA.

1 Derivation of the equation system (Eq. (17) - (20) in the main article)

We briefly derive the equation system that describes the x-component of the electric field E_x and the y-component of the magnetic field H_y of the electromagnetic waves inside and outside the phased parallel-plate waveguide array (PPWA). Region I corresponds to the space of the incident and back diffracted wave, region II refers to the PPWA space and region III to the area after transmission through the PPWA, as illustrated in Fig. 1. As in the main article, $E_{i,x}$ and $E_{i,z}$ with $i = I, III$ describe the electric field x- and z-component in the ith region, $E_{II,m,x}$ and $E_{II,m,z}$ the electric field x- and z-component in the mth waveguide in region II, $H_{i,y}$ with $i = I, III$ the magnetic field y-component in the ith region, $H_{II,m,y}$ the magnetic field y-component in the mth waveguide in region II, n_m the refractive index of the mth waveguide in a single cell, η_i with $i = I, III$ the wave impedance in region i, $\eta_{II,m}$ the wave impedance in the mth waveguide in region II, L the width of a cell, w the waveguide width, h the waveguide length, r_l the reflection coefficient for the lth order back diffracted wave at the boundary at $z = 0$, t_l the transmission coefficient for the lth order forward diffracted wave at the boundary at $z = h$, k_x and k_z the x- and z-component of the vacuum wave vector of the incident wave, k_0 the magnitude of the vacuum wave vector of the incident wave, $k_{x,l}$ and $k_{z,l}$ the x- and z-component of the vacuum wave vector of the lth order diffracted wave, $\text{sinc}(x) = \sin(x)/x$ the sinc-function, δ_{ij} the Kronecker delta, and C_m^+ and C_m^- the complex amplitudes of the forward and backward propagating waves in the mth waveguide.

In the following, we consider the boundary conditions for the tangential component E_x of the electric field and the tangential component H_y of the magnetic field at $z = 0$ and $z = h$, see Fig. 1. At this point it must be noted that we cannot obtain an analytic solution if we strictly uphold the boundary condition of matching tangential components for each single point at the boundary of each waveguide aperture. In order to obtain an analytic solution, it is necessary to approximate the boundary conditions for the tangential component in such a way as to ensure that the average value of the tangential component of the electric and magnetic fields match along the boundary of each waveguide aperture. Although this loosened requirement does not strictly match the exact boundary conditions, we obtain good agreement between the numerical simulation of the electromagnetic waves and the analytic calculation, as shown later by comparison.

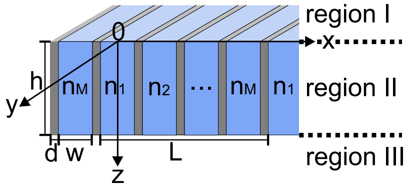

Figure 1. Schematic of the PPWA for analytic description

E_x at z=0

We assume a perfect electric conductor for the waveguide walls, which implies that E_x must equal zero at the waveguide walls at $z = 0$. Along the waveguide aperture, the condition $E_{II,m,x} = E_{I,x}$ must be fulfilled for continuity. Equation (5) contains the approximation step for the boundary condition by averaging the tangential component of the electric field over the waveguide aperture.

$$E_{I,x} = \begin{cases} 0 & \text{for x on metal} \\ E_{II,m,x} & \text{for x on m}^{\text{th}} \text{ PPW} \end{cases} \tag{1}$$

$$\Rightarrow \frac{k_z}{k_0}\exp(\mathrm{i}k_x x)\exp(\mathrm{i}k_z z) - \sum_{l=-\infty}^{+\infty}\frac{k_{z,l}}{k_0} r_l \exp(\mathrm{i}k_{x,l}x)\exp(-\mathrm{i}k_{z,l}z)$$
$$= \left.\begin{cases} 0 & \text{for x on metal} \\ C_m^+\exp(\mathrm{i}k_0 n_m z) + C_m^-\exp(-\mathrm{i}k_0 n_m z) & \text{for x on m}^{\text{th}} \text{ PPW} \end{cases}\right|_{z=0} \tag{2}$$

$$\Rightarrow \frac{k_z}{k_0}\exp(\mathrm{i}k_x x) - \sum_{l=-\infty}^{+\infty}\frac{k_{z,l}}{k_0} r_l \exp(\mathrm{i}k_{x,l}x)$$
$$= \left.\begin{cases} 0 & \text{for x on metal} \\ C_m^+ + C_m^- & \text{for x on m}^{\text{th}} \text{ PPW} \end{cases}\right| \times \exp(-\mathrm{i}k_{x,r}x) \tag{3}$$

$$\Rightarrow \frac{k_z}{k_0}\exp(\mathrm{i}\,(k_x - k_{x,r})\,x) - \sum_{l=-\infty}^{+\infty}\frac{k_{z,l}}{k_0} r_l \exp[\mathrm{i}\left(k_{x,l} - k_{x,r}\right)x]$$
$$= \left.\begin{cases} 0 & \text{for x on metal} \\ (C_m^+ + C_m^-)\exp(-\mathrm{i}k_{x,r}x) & \text{for x on m}^{\text{th}} \text{ PPW} \end{cases}\right| \int_{-\frac{w}{2}}^{-\frac{w}{2}+L} dx \text{ Integration over the cell} \tag{4}$$

$$\Rightarrow \int_{-\frac{w}{2}}^{-\frac{w}{2}+L}\frac{k_z}{k_0}\exp[\mathrm{i}\,(k_x - k_{x,r})\,x]dx - \sum_{l=-\infty}^{+\infty}\frac{k_{z,l}}{k_0} r_l \int_{-\frac{w}{2}}^{-\frac{w}{2}+L}\exp[\mathrm{i}\left(k_{x,l} - k_{x,r}\right)x]dx$$
$$= \sum_{m=1}^{M}\left(C_m^+ + C_m^-\right)\left.\int_{-\frac{w}{2}+(m-1)(w+d)}^{+\frac{w}{2}+(m-1)(w+d)}\exp(-\mathrm{i}k_{x,r}x)dx\right| \text{Integration} \tag{5}$$

$$\Rightarrow \delta_{0r}\frac{k_z}{k_0}L - \sum_{l=-\infty}^{+\infty}\frac{k_{z,l}}{k_0} r_l \delta_{lr} L = \sum_{m=1}^{M}\left(C_m^+ + C_m^-\right)\frac{2\exp[-\mathrm{i}k_{x,r}(m-1)(w+d)]\sin\frac{wk_{x,r}}{2}}{k_{x,r}} \tag{6}$$

$$\Rightarrow \delta_{0r}\frac{k_z}{k_0}L - \frac{k_{z,r}}{k_0} r_r L = \sum_{m=1}^{M}\left(C_m^+ + C_m^-\right) w\exp[-\mathrm{i}k_{x,r}(m-1)(w+d)]\operatorname{sinc}\frac{wk_{x,r}}{2} \tag{7}$$

$$\Rightarrow \delta_{0r}\frac{k_z}{k_0} - \frac{k_{z,r}}{k_0} r_r = \frac{w}{L}\sum_{m=1}^{M}\left(C_m^+ + C_m^-\right) A_{r,m}^- \tag{8}$$

with $A_{r,m}^- = \exp[-\mathrm{i}k_{x,r}(m-1)(w+d)]\operatorname{sinc}\frac{wk_{x,r}}{2}$

$$\Rightarrow r_r = \frac{\delta_{0r}k_z - \frac{w}{L}\sum_{m=1}^{M}(C_m^+ + C_m^-)A_{r,m}^- k_0}{k_{z,r}} \tag{9}$$

By changing the index of the diffraction order from r to l, we obtain the same nomenclature as in the primary manuscript:

$$r_l = \frac{\delta_{0l}k_z - \frac{w}{L}\sum_{m=1}^{M}(C_m^+ + C_m^-)A_{l,m}^- k_0}{k_{z,l}} \tag{10}$$

H_y at z=0

As above, we assume a perfect electric conductor for the waveguide walls, which implies that H_y must be continuous at the waveguide walls at $z = 0$. Along the waveguide aperture, the condition $H_{II,m,y} = H_{I,y}$ must also be fulfilled for continuity. Equation (14) contains the approximation step for the boundary condition by averaging the tangential component of the magnetic field over the waveguide aperture.

$$H_{I,y} = H_{II,m,y} \quad \text{for x in the m}^{\text{th}} \text{ PPW} \tag{11}$$

$$\Rightarrow \frac{1}{\eta_I}\exp(\mathrm{i}k_x x)\exp(\mathrm{i}k_z z) + \frac{1}{\eta_I}\sum_{l=-\infty}^{+\infty} r_l \exp(\mathrm{i}k_{x,l}x)\exp(-\mathrm{i}k_{z,l}z)$$
$$= \frac{1}{\eta_{II,m}}\left[C_m^+\exp(\mathrm{i}k_0 n_m z) - C_m^-\exp(-\mathrm{i}k_0 n_m z)\right]\Bigg|_{z=0} \tag{12}$$

$$\Rightarrow \exp(\mathrm{i}k_x x) + \sum_{l=-\infty}^{+\infty} r_l \exp(\mathrm{i}k_{x,l}x)$$
$$= \frac{\eta_I}{\eta_{II,m}}\left(C_m^+ - C_m^-\right)\Bigg| \int_{-\frac{w}{2}+(m-1)(w+d)}^{+\frac{w}{2}+(m-1)(w+d)} dx \quad \text{Integration over m}^{\text{th}} \text{ PPW} \tag{13}$$

$$\Rightarrow \int_{-\frac{w}{2}+(m-1)(w+d)}^{+\frac{w}{2}+(m-1)(w+d)} \exp(\mathrm{i}k_x x)dx + \sum_{l=-\infty}^{+\infty} r_l \int_{-\frac{w}{2}+(m-1)(w+d)}^{+\frac{w}{2}+(m-1)(w+d)} \exp(\mathrm{i}k_{x,l}x)dx$$
$$= \frac{\eta_I}{\eta_{II,m}} w\left(C_m^+ - C_m^-\right)\Bigg| \text{Integration} \tag{14}$$

$$\Rightarrow \frac{2\exp[\mathrm{i}k_x(m-1)(w+d)]\sin\frac{wk_x}{2}}{k_x} + \sum_{l=-\infty}^{+\infty} r_l \frac{2\exp[\mathrm{i}k_{x,l}(m-1)(w+d)]\sin\frac{wk_{x,l}}{2}}{k_{x,l}}$$
$$= \frac{\eta_I}{\eta_{II,m}} w\left(C_m^+ - C_m^-\right) \tag{15}$$

$$\Rightarrow \exp[\mathrm{i}k_x(m-1)(w+d)]\operatorname{sinc}\frac{wk_x}{2} + \sum_{l=-\infty}^{+\infty} r_l \exp[\mathrm{i}k_{x,l}(m-1)(w+d)]\operatorname{sinc}\frac{wk_{x,l}}{2}$$
$$= \frac{\eta_I}{\eta_{II,m}}\left(C_m^+ - C_m^-\right) \tag{16}$$

$$\Rightarrow A_{0,m}^+ + \sum_{l=-\infty}^{+\infty} r_l A_{l,m}^+ = \frac{\eta_I}{\eta_{II,m}}\left(C_m^+ - C_m^-\right) \tag{17}$$

$$\text{with } A_{l,m}^+ = \exp[\mathrm{i}k_{x,l}(m-1)(w+d)]\operatorname{sinc}\frac{wk_{x,l}}{2}$$

$$\Rightarrow \sum_{l=-\infty}^{+\infty} r_l A_{l,m}^+ = \frac{\eta_I}{\eta_{II,m}}\left(C_m^+ - C_m^-\right) - A_{0,m}^+ \tag{18}$$

In analogy to the previous evaluation of the boundary conditions at $z = 0$, we calculate E_x and H_y at $z = h$. Here, Eqs. (23) and (31) contain the averaging process.

E_x at z=h

$$E_{III,x} = \begin{cases} 0 & \text{for x on metal} \\ E_{II,m,x} & \text{for x on m}^{\text{th}} \text{ PPW} \end{cases} \tag{19}$$

$$\Rightarrow \sum_{l=-\infty}^{+\infty} t_l \exp(\mathrm{i}k_{x,l}x)\exp[\mathrm{i}k_{z,l}(z-h)]\frac{k_{z,l}}{k_0}$$
$$= \begin{cases} 0 & \text{for x on metal} \\ C_m^+\exp(\mathrm{i}k_0 n_m z) + C_m^-\exp(-\mathrm{i}k_0 n_m z) & \text{for x on m}^{\text{th}} \text{ PPW} \end{cases}\Bigg|_{\text{z=h}} \tag{20}$$

$$\Rightarrow \sum_{l=-\infty}^{+\infty} t_l \exp(\mathrm{i}k_{x,l}x)\frac{k_{z,l}}{k_0}$$
$$= \begin{cases} 0 & \text{for x on metal} \\ C_m^+\exp(\mathrm{i}k_0 n_m h) + C_m^-\exp(-\mathrm{i}k_0 n_m h) & \text{for x on m}^{\text{th}} \text{ PPW} \end{cases}\Bigg| \times \exp(-\mathrm{i}k_{x,r}x) \tag{21}$$

$$\Rightarrow \sum_{l=-\infty}^{+\infty} t_l \exp[\mathrm{i}\left(k_{x,l}-k_{x,r}\right)x]\frac{k_{z,l}}{k_0}$$
$$= \begin{cases} 0 & \text{for x on metal} \\ [C_m^+\exp(\mathrm{i}k_0 n_m h) + C_m^-\exp(-\mathrm{i}k_0 n_m h)]\exp(-\mathrm{i}k_{x,r}x) & \text{for x on m}^{\text{th}} \text{ PPW} \end{cases}\Bigg| \int_{-\frac{w}{2}}^{-\frac{w}{2}+L} dx \tag{22}$$

$$\Rightarrow \sum_{l=-\infty}^{+\infty} t_l \frac{k_{z,l}}{k_0} \int_{-\frac{w}{2}}^{-\frac{w}{2}+L} \exp[\mathrm{i}\left(k_{x,l}-k_{x,r}\right)x]dx$$
$$= \sum_{m=1}^{M}\left[C_m^+\exp(\mathrm{i}k_0 n_m h) + C_m^-\exp(-\mathrm{i}k_0 n_m h)\right] \int_{-\frac{w}{2}+(m-1)(w+d)}^{+\frac{w}{2}+(m-1)(w+d)} \exp(-\mathrm{i}k_{x,r}x)dx \Bigg| \text{Integration} \tag{23}$$

$$\Rightarrow \sum_{l=-\infty}^{+\infty} t_l L \delta_{l,r}\frac{k_{z,l}}{k_0} = \sum_{m=1}^{M}\left[C_m^+\exp(\mathrm{i}k_0 n_m h) + C_m^-\exp(-\mathrm{i}k_0 n_m h)\right] wA_{r,m}^- \tag{24}$$

with $A_{r,m}^- = \exp[-\mathrm{i}k_{x,r}(m-1)(w+d)]\mathrm{sinc}\frac{wk_{x,r}}{2}$

$$\Rightarrow t_r L\frac{k_{z,r}}{k_0} = \sum_{m=1}^{M}\left[C_m^+\exp(\mathrm{i}k_0 n_m h) + C_m^-\exp(-\mathrm{i}k_0 n_m h)\right] wA_{r,m}^- \tag{25}$$

$$\Rightarrow t_r = \frac{w}{L}\frac{k_0}{k_{z,r}}\sum_{m=1}^{M}\left[C_m^+\exp(\mathrm{i}k_0 n_m h) + C_m^-\exp(-\mathrm{i}k_0 n_m h)\right] A_{r,m}^- \tag{26}$$

By changing the index of the diffraction order from r to l, we obtain the same nomenclature as in the primary manuscript:

$$t_l = \frac{w}{L}\frac{k_0}{k_{z,l}}\sum_{m=1}^{M}\left[C_m^+\exp(\mathrm{i}k_0 n_m h) + C_m^-\exp(-\mathrm{i}k_0 n_m h)\right] A_{l,m}^- \tag{27}$$

H_y **at z=h**

$$
\begin{aligned}
& H_{III,y} = H_{II,m,y} \quad \text{for x in the m}^{\text{th}} \text{ PPW} && (28)\\
\Rightarrow & \frac{1}{\eta_{III}} \sum_{l=-\infty}^{+\infty} t_l \exp(\mathrm{i}k_{x,l}x)\exp[\mathrm{i}k_{z,l}(z-h)] \\
& = \frac{1}{\eta_{II,m}} \left[C_m^+ \exp(\mathrm{i}k_0 n_m z) - C_m^- \exp(-\mathrm{i}k_0 n_m z)\right]\Big|_{z=h} && (29)\\
\Rightarrow & \frac{1}{\eta_{III}} \sum_{l=-\infty}^{+\infty} t_l \exp(\mathrm{i}k_{x,l}x) \\
& = \frac{1}{\eta_{II,m}} \left[C_m^+ \exp(\mathrm{i}k_0 n_m h) - C_m^- \exp(-\mathrm{i}k_0 n_m h)\right]\Bigg| \int_{-\frac{w}{2}+(m-1)(w+d)}^{+\frac{w}{2}+(m-1)(w+d)} dx \text{ Integration over m}^{\text{th}} \text{ PPW} && (30)\\
\Rightarrow & \frac{1}{\eta_{III}} \sum_{l=-\infty}^{+\infty} t_l \int_{-\frac{w}{2}+(m-1)(w+d)}^{+\frac{w}{2}+(m-1)(w+d)} \exp(\mathrm{i}k_{x,l}x)dx \\
& = \frac{1}{\eta_{II,m}} \left[C_m^+ \exp(\mathrm{i}k_0 n_m h) - C_m^- \exp(-\mathrm{i}k_0 n_m h)\right] \int_{-\frac{w}{2}+(m-1)(w+d)}^{+\frac{w}{2}+(m-1)(w+d)} 1dx \Bigg| \text{ Integration} && (31)\\
\Rightarrow & \sum_{l=-\infty}^{+\infty} t_l w A_{l,m}^+ = \frac{\eta_{III}}{\eta_{II,m}} w \left[C_m^+ \exp(\mathrm{i}k_0 n_m h) - C_m^- \exp(-\mathrm{i}k_0 n_m h)\right] && (32)\\
& \text{with } A_{l,m}^+ = \exp[\mathrm{i}k_{x,l}(m-1)(w+d)]\operatorname{sinc}\frac{wk_{x,l}}{2} \\
\Rightarrow & \underline{\underline{\sum_{l=-\infty}^{+\infty} t_l A_{l,m}^+ = \frac{\eta_{III}}{\eta_{II,m}} \left[C_m^+ \exp(\mathrm{i}k_0 n_m h) - C_m^- \exp(-\mathrm{i}k_0 n_m h)\right]}} && (33)
\end{aligned}
$$

The relaxation of the electromagnetic boundary conditions by requiring continuity only for the average value of the tangential components of the electric and magnetic field instead of point-wise compliance can be observed by comparison between the numerically calculated electromagnetic fields in the PPWA and the corresponding analytic calculation. This is shown for the example of H_y in the PPWA in Fig. 2, where H_y is calculated analytically and numerically for $-L < z < h+L$. As can be seen in the insets, which display a zoom of the magnetic field H_y at the boundary $z=0$, the magnetic field H_y is strictly continuous in case of the numeric calculation, whereas H_y is discontinuous along the waveguide boundary in the analysis due to the averaging process described above. However, since the width of the waveguides is small compared to the wavelength of the waves, the averaging error is marginal and deviations between the numerical and analytic calculation of the tangential fields at the boundaries are almost negligible.

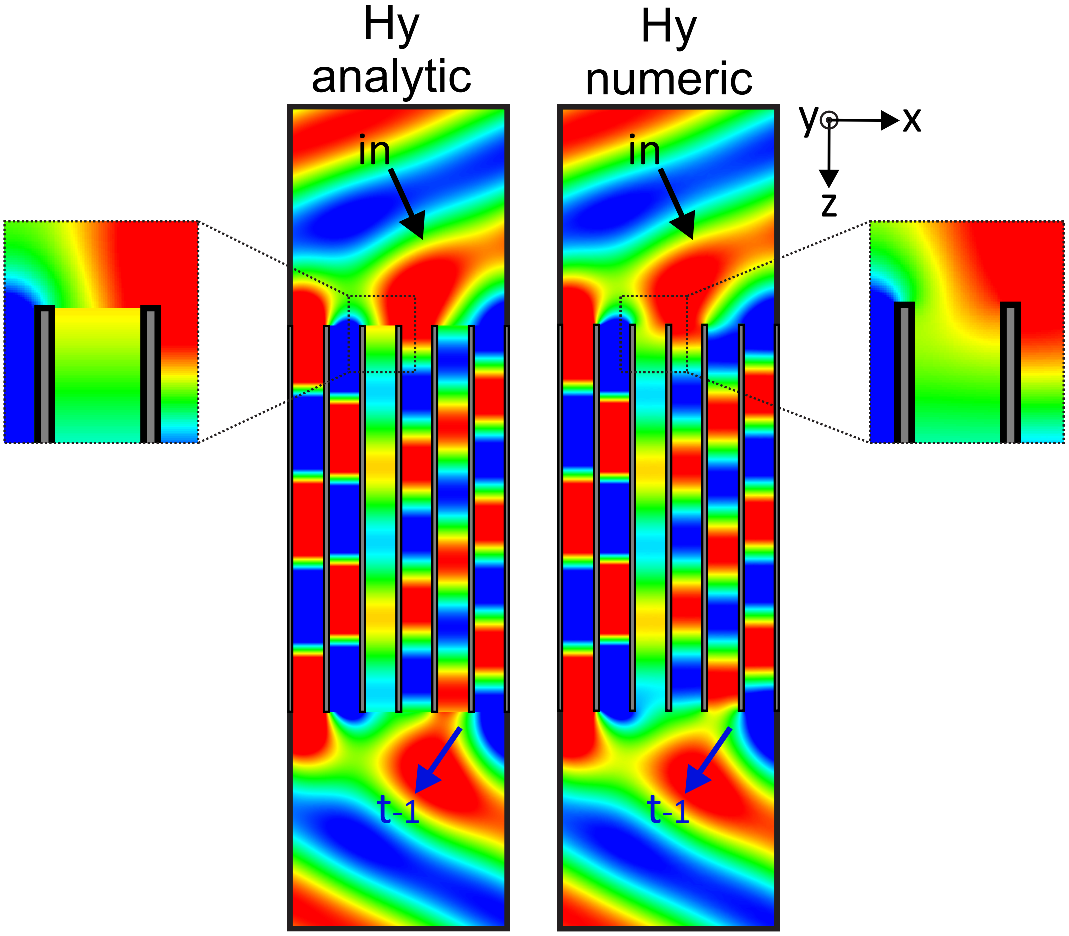

Figure 2. Comparison between the analytically calculated magnetic field H_y in the PPWA (left) and the numerically calculated magnetic field H_y (right). The insets show the zoomed magnetic field H_y at the boundary at $z = 0$.

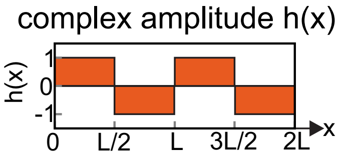

Figure 3. Function $h(x)$ of the binary phase grating.

2 Fourier Series of a 180° phase grating and an arbitrary phase and amplitude grating

As described in the main article, the impact of the PPWA on the forward wave diffraction of arbitrary waveforms at $z = h$ can be described by a superposition of an $L/2$-periodic amplitude and phase grating $g(x)$ and an L-periodic binary phase grating $h(x)$ that induces zero phase shift in the first half cycle and a 180° phase shift in the second half cycle. For the calculation of the transmitted diffraction orders at $z = h$, we develop both functions $g(x)$ and $h(x)$ in terms of a Fourier series and obtain the function of the diffracted waves as the product $f(x) = g(x) \times h(x)$.

Fourier series of an arbitrary amplitude and phase grating with a period of L/2

The Fourier series of an arbitrary $L/2$-periodic amplitude and phase grating can be readily obtained by

$$g(x) = \sum_{k=-\infty}^{+\infty} a_k \exp(\mathrm{i}k\omega_0 x) \text{ with } \omega_0 = \frac{4\pi}{L}, a_k \in \mathbb{C} \text{ and } k \in \mathbb{Z} \tag{34}$$

$$\Rightarrow g(x) = \sum_{k=-\infty}^{+\infty} a_k \exp(\mathrm{i}k4\pi/Lx) \tag{35}$$

where a_k is the complex Fourier amplitude of the amplitude and phase grating.

Fourier series of the binary phase grating

The function $h(x)$ of the binary phase grating is depicted in Fig. 3. As obvious, the binary phase grating induces a 180° phase shift in the second half cycle of the periodic function, while the amplitude remains constant at unity. With ω_0 being the (one-dimensional) wavevector, L the width of a cell and c_k the Fourier amplitude, the Fourier series of $h(x)$ is described by

$$h(x) = \sum_{k=-\infty}^{+\infty} c_k \exp(\mathrm{i}k\omega_0 x) \text{ with } \omega_0 = \frac{2\pi}{L} \text{ and } c_k = \frac{1}{L}\int_0^L h(x)\exp(-\mathrm{i}k\omega_0 x)\,\mathrm{d}x \text{ and } k \in \mathbb{Z} \tag{36}$$

$$\Rightarrow c_0 = \frac{1}{L}\int_0^L g(x)\mathrm{d}x = 0 \tag{37}$$

$$\Rightarrow c_{k,k\neq 0} = \frac{1}{L}\left[\int_0^{L/2} \exp(-\mathrm{i}k\omega_0 x)\,\mathrm{d}x - \int_{L/2}^{L} \exp(-\mathrm{i}k\omega_0 x)\,\mathrm{d}x\right] \tag{38}$$

$$\Rightarrow c_{k,k\neq 0} = \frac{\mathrm{i}}{Lk\omega_0}\left[\exp(-\mathrm{i}k\omega_0 L/2) - 1 - \exp(-\mathrm{i}k\omega_0 L) + \exp(-\mathrm{i}k\omega_0 L/2)\right] \tag{39}$$

$$\Rightarrow c_{k,k\neq 0} = \frac{\mathrm{i}}{k2\pi}\left[\exp(-\mathrm{i}k\pi) - 1 - \exp(-\mathrm{i}k2\pi) + exp(-\mathrm{i}k\pi)\right] \tag{40}$$

$$\Rightarrow c_{k,k\neq 0} = \frac{\mathrm{i}}{k2\pi}\left[2\exp(-\mathrm{i}k2\pi) - 2\right] \tag{41}$$

$$\Rightarrow c_{k,k\neq 0} = \frac{\mathrm{i}}{k\pi}\left[\exp(-\mathrm{i}k2\pi) - 1\right] \tag{42}$$

$$\Rightarrow c_{k,k\neq 0} = \begin{cases} 0 & \text{for k} = \text{even} = \pm 2, \pm 4, \ldots \\ \frac{-2\mathrm{i}}{k\pi} & \text{for k} = \text{odd} = \pm 1, \pm 3, \ldots \end{cases} \tag{43}$$

$$\Rightarrow h(x) = \sum_{k=-\infty,\, k=\text{odd}}^{+\infty} \frac{-2\mathrm{i}}{k\pi} \exp(\mathrm{i}k2\pi/Lx) \tag{44}$$

$$\Rightarrow h(x) = \sum_{k'=-\infty}^{+\infty} \frac{-2\mathrm{i}}{k'\pi} \exp\left[\mathrm{i}(2k'+1)2\pi/Lx\right] \text{ with } k = 2k'+1 \text{ and } k' \in \mathbb{Z} \tag{45}$$
